# Colonization with two different *Blastocystis* subtypes in DSS-induced colitis mice is associated with strikingly different microbiome and pathological features

**DOI:** 10.7150/thno.81583

**Published:** 2023-02-05

**Authors:** Lei Deng, Lukasz Wojciech, Chin Wen Png, Dorinda Yan Qin Kioh, Yuxiang Gu, Thet Tun Aung, Benoit Malleret, Eric Chun Yong Chan, Guangneng Peng, Yongliang Zhang, Nicholas Robert John Gascoigne, Kevin Shyong Wei Tan

**Affiliations:** 1 Laboratory of Molecular and Cellular Parasitology, Department of Microbiology and Immunology, Healthy Longevity Translational Research Programme, Yong Loo Lin School of Medicine, National University of Singapore, 5 Science Drive 2, Singapore 117545, Singapore; 2 Department of Microbiology and Immunology, Immunology Translational Research Programme, Yong Loo Lin School of Medicine, National University of Singapore, 5 Science Drive 2, Singapore 117545, Singapore; 3 Department of Pharmacy, Faculty of Science, National University of Singapore, 18 Science Drive 4, Singapore 117559, Singapore; 4 The Key Laboratory of Animal Disease and Human Health of Sichuan Province, College of Veterinary Medicine, Sichuan Agricultural University, Chengdu, Sichuan, 611130, China

**Keywords:** *Blastocystis*, Gut microbiota, IBD, DSS-induced colitis, Short-chain fatty acids

## Abstract

**Rationale:** The gut microbiota plays a significant role in the pathogenesis of inflammatory bowel disease (IBD). However, the role of *Blastocystis* infection and *Blastocystis*-altered gut microbiota in the development of inflammatory diseases and their underlying mechanisms are not well understood.

**Methods:** We investigated the effect of *Blastocystis* ST4 and ST7 infection on the intestinal microbiota, metabolism, and host immune responses, and then explored the role of *Blastocystis*-altered gut microbiome in the development of dextran sulfate sodium (DSS)-induced colitis in mice.

**Results:** This study showed that prior colonization with ST4 conferred protection from DSS-induced colitis through elevating the abundance of beneficial bacteria, short-chain fatty acid (SCFA) production and the proportion of Foxp3^+^ and IL-10-producing CD4^+^ T cells. Conversely, prior ST7 infection exacerbated the severity of colitis by increasing the proportion of pathogenic bacteria and inducing pro-inflammatory IL-17A and TNF-α-producing CD4^+^ T cells. Furthermore, transplantation of ST4- and ST7-altered microbiota resulted in similar phenotypes.

**Conclusions:** Our data showed that ST4 and ST7 infection exert strikingly differential effects on the gut microbiota, and these could influence the susceptibility to colitis. ST4 colonization prevented DSS-induced colitis in mice and may be considered as a novel therapeutic strategy against immunological diseases in the future, while ST7 infection is a potential risk factor for the development of experimentally induced colitis that warrants attention.

## Introduction

Inflammatory bowel disease (IBD) is a chronic gastrointestinal (GI) disorder, including Crohn's disease (CD) and ulcerative colitis (UC) 1. Numerous factors are thought to play important roles in the pathogenesis of IBD, including the gut microbiome and immune dysregulation 2, 3. The gut microbiome is composed of trillions of indigenous bacteria, archaea, fungi, viruses, and protists 4. Studies have primarily focused on associations between bacteria and IBD, while the influence of gut protists on this disease is largely unexplored 5. Several protists are typically known for causing dysenteric infections, such as *Entamoeba histolytica*, and *Cryptosporidium* spp. 6, 7. However, the impact of other protists on host health remains debated, particularly for *Blastocystis*. *Blastocystis* is the most common gut protist found in humans and animals, and harbors extensive genetic heterogeneity 8. The relationship between* Blastocystis* and IBD is controversial. Several studies have shown that *Blastocystis* prevalence was higher in IBD patients than in healthy individuals 9, 10, while *Blastocystis* was also considered to be an enteric commensal as it was found to be more abundant in asymptomatic individuals and associated with increased gut bacterial diversity 11.

*Blastocystis* colonization or infection could modulate the gut microbial composition and may influence the progression of IBD 12. Experimental colitis induced by dextran sulfate sodium (DSS) in rodents is a common animal model of human IBD research 13. Our previous study showed that infection with *Blastocystis* ST7 exacerbated the severity of DSS-induced colitis in a mouse model. This was accompanied with decreased proportions of beneficial bacteria such as *Bifidobacterium* and *Lactobacillus* 14. However, our recent study showed that colonization with *Blastocystis* ST4 can reduce the severity of colitis through modulating the gut microbiota and activation of T helper-2 (Th2) and regulatory T cell (Treg) immune responses 15. These earlier studies involved inoculation of *Blastocystis* after DSS treatment and investigated the effects of *Blastocystis* infection or colonization in promoting recovery or exacerbating DSS-induced colitis. However, whether prior *Blastocystis* infection has an impact on subsequent experimentally induced colitis and whether *Blastocystis*-altered gut microbiota is also able to influence the development of IBD remains unexplored.

Although several studies have shown the associations between *Blastocystis* colonization and host health, most studies do not demonstrate whether the effect of *Blastocystis* on host health is a direct result of immune regulation or an indirect effect of microbiome modulation. On another hand, studies looking at the effect of *Blastocystis* on the gut microbiota employed the use of one subtype, although it has been reported that distinct subtypes interact differently with bacteria. In this study, we compared the effects of colonization by two *Blastocystis* subtypes, ST4 and ST7, available as axenic cultures (pure cultures grown in the absence of bacteria) that have shown distinct effects on gut epithelial barrier function and immune responses in several* in vitro* studies 16, 17. ST4 and ST7 were originally identified in a Wistar rat and a patient with gastrointestinal symptoms, respectively 18, 19. We observed that prior ST4 colonization can prevent induced colitis when mice were later challenged with DSS, through elevating the proportion of beneficial bacteria, SCFAs, and protective immune responses. In contrast, prior infection with ST7 exacerbated colitis through inducing pro-inflammatory Th1 and Th17 immune responses. These results provide evidence that distinct modifications of gut microbial composition are triggered by specific *Blastocystis* subtypes, and that *Blastocystis*-altered gut microbiota play roles in the development of experimental colitis.

## Materials and methods

### Blastocystis cultures

Axenized cultures of *Blastocystis* ST4 and ST7 were used in this study. ST4-WR1 was first identified in a healthy Wistar rat in National University of Singapore (NUS), Singapore 19. ST7-B was originally isolated from a diarrheal patient at the Singapore General Hospital in the early 1990s before the Institutional Review Board was established at the NUS 18. *Blastocystis* was maintained in 10 ml of pre-reduced Iscove's modified Dulbecco's medium (IMDM) (Gibco) supplemented with heat-inactivated 10% horse serum (Gibco), and NaHCO_3_. *Blastocystis* cells were cultured in an Anaerojar (Oxoid) with Oxoid CO_2_ Gen Sachet (Thermo Scientific) at 37 °C. A hemocytometer (Kova International) was used to calculate the number of *Blastocystis* cells.

### Mice and treatment

C57BL/6 mice, aged 8-12 weeks and bred in-house, were maintained in the animal facility of the National University of Singapore (NUS). Littermates of the same sex and age were randomly assigned to the different experimental groups. All animal experiments were performed under the Singapore National Advisory Committee for Laboratory Animal Research guidelines and approved by the Institutional Animal Care and Use Committee of NUS (protocol no. R19-1259).

Oral infection of *Blastocystis*. Mice (n = 6 per group) were orally gavaged with 5 × 10^7^ live *Blastocystis* ST4 and ST7 cells suspended in sterile phosphate-buffered saline (PBS). The control mice (n = 6) were orally gavaged with equal amounts of PBS at the same time. The mice used in all the experiments were age and sex matched. Each treatment was housed together in two cages (n = 3).

DSS-induced colitis. Mice were exposed to 2% DSS (molecular mass = 36,000-50,000 Da; MP Biomedicals) w/v in drinking water for 7 days. The weight, stool consistency, and presence of fecal blood were recorded each day. The disease activity index (DAI) was used to determine the severity of colitis as previously described 20.

Antibiotic administration. Mice were administrated with a broad-spectrum antibiotic cocktail ampicillin (1 g/L), vancomycin (500 mg/L), neomycin sulfate (1 g/L), and metronidazole (1 g/L) in drinking water for 14 days 21, before the microbiota transplant experiments.

### Microbiota transfer

To exclude the effect of *Blastocystis* on the severity of colitis, feces collected from ST4 and ST7 infected mice and non-infected control mice were frozen at -80°C, and then thawed in pre-reduced PBS. This procedure ruptures *Blastocystis* cells, and the absence of live *Blastocystis* cells was confirmed by culturing the feces in Jones' medium. Fecal microbiota transplantation (FMT) was performed as described 22. In brief, frozen feces were pooled from control, ST4-colonized, and ST7-infected mice, respectively, diluted with pre-reduced PBS (50 mg/ml), and administered to recipient mice by oral gavage (10 mg/mice) three times a week for one week.

### Scanning electron microscopy

Scanning electron microscopy (SEM) was performed as previously described 23. In brief, the cecum/colon tissues were collected and then processed by opening the gut longitudinally without disturbing the intestinal contents. The opened tissues were pinned down to a silicone mat in four corners and fixed in 2.5% glutaraldehyde at 4°C overnight, and then washed twice with PBS. The washed tissues were kept at 4°C until further processing. The tissues were processed by post-fixing in 1% osmium tetroxide for 1 h, and then dehydrated first in 25% ethanol for 5 minutes followed by 50%,75% and 95% ethanol for 10 minutes each. After which, the sample was fixed in 100% ethanol for 10 minutes (3 changes). The dried samples were coated with 25 nm of gold and imaged on a field emission JSM-6701F SEM at a voltage of 10 kV.

### DNA extraction and real-time quantitative PCR

Total genomic DNA from feces of control, ST4-colonized, and ST7-infected mice at day 0 (before *Blastocystis* inoculation), and day 14 (end of experiment) was extracted using QIAamp Fast DNA Stool Mini Kit (Qiagen) according to the manufacturer's protocol. Real-time quantitative PCR (qPCR) was used to estimate the number of *Blastocystis* cells in fecal samples at the end point as described 24.

### Colon histology

Colonic tissues were collected at the end of each experiment and fixed in 4% neutral buffered formalin, and then processed and embedded in paraffin. Sections of 4.5 μm were prepared and stained with hematoxylin and eosin (H&E). Histology scoring was performed in a blinded fashion, whereby changes in intestinal crypt architecture, degree of inflammation, and epithelium damage were scored as previously described 25. A combined score of crypt architecture, tissue damage, goblet cell loss, and inflammatory cell infiltration was determined as follows: crypt architecture: score 0, normal; 1, irregular; 2, moderate crypt loss (10-50%); 3, severe crypt loss (50-90%); 4, small/medium sized ulcers (<10 crypt widths); 5, large ulcers (>10 crypt widths). Tissue damage: score 0, no damage; 1, discrete lesions; 2, mucosal erosions; 3, extensive mucosal damage. Goblet cell loss: score 0, normal; 1, 10-25% loss; 2, 25-50% loss; 3, = >50% loss. Inflammatory cell infiltration: score 0, occasional infiltration; 1, increasing leukocytes in lamina propria; 2, confluence of leukocytes extending to submucosa; 3, transmural extension of inflammatory infiltrates.

### 16S rRNA gene sequencing and bioinformatics analysis

Genomic DNA was extracted from fecal samples. The 16S rRNA gene sequencing library was constructed using a MetaVX Library Preparation Kit (South Plainfield, NJ). Briefly, 20-30 ng of DNA was used to generate amplicons that cover V3 and V4 hypervariable regions of the 16S rRNA gene of bacteria. The forward primer contains the sequence 'CCTACGGRRBGCASCAGKVRVGAAT', and the reverse primers contain the sequence 'GGACTACNVGGGTWTCTAATCC'. The 25 µl PCR mixture was prepared with 2.5 µl of TransStart buffer, 2 µl of dNTPs, 1 µl of each primer, 0.5 µl of TransStart Taq DNA polymerase, and 20 ng template DNA. PCR was performed by the following program: 3 min of denaturation at 94℃, 24 cycles of 5 s at 95℃, 90 s of annealing at 57℃, 10 s of elongation at 72℃, and a final extension at 72℃ for 5 min. Indexed adapters were added to the ends of the amplicons by limited cycle PCR. Finally, the library was purified using magnetic beads. DNA concentration was determined with a microplate reader (Tecan, Infinite 200 Pro) and the expected ~600 bp fragment size was determined by 1.5% agarose gel electrophoresis. Next generation sequencing was conducted on an Illumina Novaseq Platform (Illumina, San Diego, USA) at Azenta Inc (USA). Automated cluster generation and 250 paired-end sequencing with dual reads were performed according to the manufacturer's instructions.

Paired-end sequences of positive and negative reads were filtered, denoised, and had chimeras removed to obtain amplicon sequence variants (ASVs) through DADA2 using Quantitative Insights into Microbial Ecology 2 (Qiime2) plugin 26. The ribosomal database program (RDP) Classifier Bayes algorithm was trained using Silva 138 database to perform a taxonomic analysis 27. Shannon and Chao1 were used to estimate the bacterial diversity and richness respectively. Beta diversity was assessed by permutational multivariate analysis of variance (PERMANOVA). Principal coordinates analysis (PCoA) plots were constructed based on Bray-Curtis dissimilarity to illustrate the differences in community structure between different groups. Heatmaps were used to show the different taxa between groups. Linear discriminant analysis effect size (LEfSe) analysis was performed to detect bacterial taxa with significantly different abundance among different groups with *P*-value <0.05 and LDA score >4 (https://huttenhower.sph.harvard.edu/galaxy/).

### RNA sequencing and bioinformatics analysis

Colon tissues were collected and washed with PBS to discard the luminal contents, and then dissected into 1 cm pieces for RNA extraction. RNA was extracted using RNAzol RT (Sigma-Aldrich) according to the manufacturer's protocol. One μg total RNA was used for the library preparation. Poly(A) mRNA isolation was performed using Oligo (dT) beads. The mRNA fragmentation was performed using divalent cations and high temperatures. Priming was performed using Random Primers (Invitrogen). First-strand cDNA and the second-strand cDNA were synthesized. The purified double-stranded cDNA was then treated to repair both ends and a dA-tailing in one reaction, followed by a T-A ligation to add adaptors to both ends was performed. Size selection of Adaptor-ligated DNA was then performed using MagMAX™ DNA Multi-Sample Ultra Kit (Applied Biosystems). Each sample was then amplified by PCR using P5 ('AATGATACGGCGACCACCGA') and P7 ('CAAGCAGAAGACGGCATACGAGAT') primers and the PCR products were validated using Agilent 2100. Then libraries with different indexes were multiplexed and loaded on an Illumina Novaseq6000 instrument at Azenta Inc (USA).

To remove adapters, primers, or fragments thereof, and quality of bases lower than 20, pass filter data of fastq format were processed by Cutadapt (V4.1, phred cutoff: 20, error rate: 0.1, adapter overlap: 1bp, min. length: 75, proportion of N: 0.1) to give high-quality clean data. Reference genome sequences and gene model annotation files were downloaded from ENSEMBL (https://asia.ensembl.org/index.html). Hisat2 (v2.1.0) was used to index reference genome sequences 28. Principal component analysis (PCA) was generated by corrplot package in R language. Differential expression analysis was done using the Limma Bioconductor package 29. Padj of genes was set at < 0.05 to detect differential expressed genes (DEGs). GOSeq (v1.34.1) was used to identify GO terms that annotate a list of enriched genes with a significant padj less than 0.05. KEGG (Kyoto Encyclopedia of Genes and Genomes) is a collection of databases dealing with genomes, biological pathways, diseases, drugs, and chemical substances (http://en. wikipedia.org/wiki/KEGG). We used in-house scripts to enrich significant differential expression genes in KEGG pathways. Gene Set Enrichment Analysis (GSEA) was generated in GSEABase package in R 30, 31.

### LC/MS/MS assay

Liquid chromatography/tandem mass spectrometry (LC/MS/MS) was carried out for analysis of short-chain fatty acids (SCFAs) in derivatized stool extracts as previously described 32. Briefly, 500 μl of ice-cold extraction solvent containing 10 μM of d_5_-benzoic acid as internal standard (IS) was added to 250 mg of fecal sample and subjected to vortex mixing for 5 min at ambient temperature. The suspension was then centrifuged at 18,000*g* for 10 min at 4 °C. The supernatant was carefully removed and centrifuged again at 18,000*g* for 10 min at 4 °C. An aliquot of 100 μl was subsequently derivatized using a final concentration of 10 mM aniline and 5 mM EDC for 2 h at 4 °C. The derivatization reaction was quenched using a final concentration of 18 mM succinic acid and 4.6 mM 2-mercaptoethanol for 2 h at 4 °C. All samples were stored at 4 °C until analysis on the same day. Analysis was performed using an Agilent 1290 Infinity LC system (Agilent Technologies) interfaced with an AB Sciex QTRAP 5500 hybrid linear ion-trap quadrupole mass spectrometer equipped with a TurboIonSpray source (Applied Biosystems).

### Isolation of colon lamina propria cells

To analyze colonic lymphocytes, the colon tissues were longitudinally opened and washed with ice-cold PBS to remove luminal contents. The tissues were cut into 1 cm pieces and incubated in Roswell Park Memorial Institute (RPMI) 1640 medium (Sigma-Aldrich) containing 1 mM EDTA (Sigma-Aldrich) and 1 mM DTT (Sigma-Aldrich) at room temperature for 20 min under slow rotation and spun down to remove the supernatant. The remaining tissue pieces were incubated in RPMI containing 25% HEPES, 10% fetal calf serum (FCS), 1 mM EDTA, and 1 mM DTT at 37 ℃ for 1 h under slow rotation and then washed by PBS to remove epithelial cells and intraepithelial lymphocytes. Tissue pieces were digested with Liberase (Sigma-Aldrich) at 37 ℃ for 30 min under slow rotation. The digested tissue pieces and supernatants were filtered by 70 μm cell strainer and glass wool separately. After centrifugation, pellets containing the LP lymphocytes were harvested.

### Flow cytometry analysis

Lymphocytes were stimulated for 6 h with a cell stimulation cocktail of PMA (50 ng/ml), ionomycin (750 ng/ml), and GolgiStop (monensin, BD Biosciences). Live/dead stain was used to evaluate the viability of the cells using live/dead fixable viability stain kits. For surface staining, stimulated cells were stained with TCR beta monoclonal antibody (BUV510, eBioscience), and anti-CD4 (BUV395; Biolegend). Fixation and permeabilization buffers from Biolegend were used for intracellular cytokine staining. Fixed and permeabilized cells were stained with fluorochrome-conjugated anti-mouse antibodies against interleukin (IL)-4 (BUV421; Biolegend), IL-10 (PE; Biolegend), IL-17A (APC; eBioscience), interferon-gamma (IFN-γ) (BUV711; Biolegend), and tumor necrosis factor (TNF-α) (APC; eBioscience) at 4 ℃ for 10 h. Flow cytometry analysis was performed on Fortessa X-20 (BD biosciences) and the data were analyzed using FlowJo_V10 software.

### Statistical analysis

All statistical analysis was performed using Prism 8 (Graphpad Software Inc.). Two independent experiments were performed. For comparisons of two groups, Student's two-tailed t-test was used. When three or more groups were analyzed, one-way or two-way ANOVA with multiple comparisons test was used. Error bars on graphs display the mean and SEM. *P* values of < 0.05 were considered significant; the following symbols were used to indicate significance levels: *, *P* < 0.05; **, *P* < 0.01; ***, *P* < 0.001; and ****, *P* < 0.0001.

## Results

### Effects of *Blastocystis* ST4 and ST7 infection on bacterial compositions

**Figure 1:**
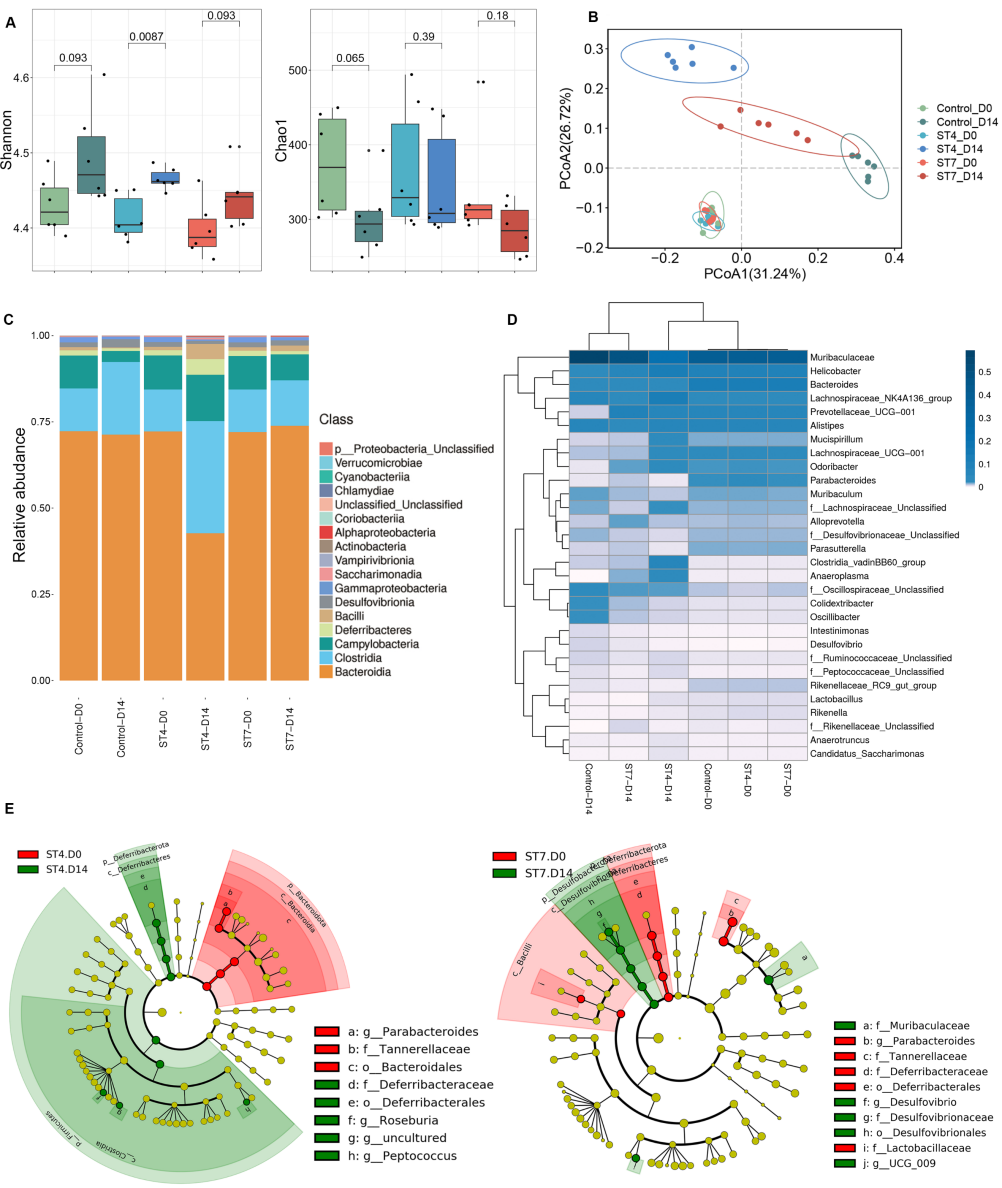
***Blastocystis* ST4 and ST7 infection caused differential gut microbial signatures**. (**A**) Alpha diversity was measured by Shannon and Chao1 indices (n = 6). (**B**) Principal co-ordinates analysis (PCoA) based on Bray-Curtis dissimilarities of fecal gut microbiota derived from control (green dots), ST4 (blue dots), and ST7 (red dots) mice at day 0 and day 14. (**C**) Relative abundance of the different taxa at the class level. (**D**) Heatmap showing *Blastocystis* ST4 and ST7-associated taxonomic markers at day 14. (**E**) LefSe analysis showed the differentially abundant bacterial taxa between ST4 and ST7 groups relative to the baseline. Data are representative of two independent experiments and shown as the mean ± SEM.

C57BL/6 mice were orally gavaged with *Blastocystis* ST4 and ST7 three times a week for two weeks (Figure S1A). The number of* Blastocystis* cells and colonization status was confirmed by qPCR and scanning electron microscopy (SEM) (Figure S1B-C). Although the bacterial richness reflected by Chao1 did not show significant differences among the three groups, the Shannon index, reflecting the bacterial diversity, was significantly increased in the ST4-colonized mice (Figure 1A). ST7 effects on bacterial diversity were comparable to those in fecal samples at day 0 (Figure 1A). We next performed PCoA analysis based on Bray-Curtis dissimilarities to investigate the effect of *Blastocystis* colonization on microbial community structure (β-diversity). At baseline (day 0), the ST4, ST7, and control mice showed similar microbiota profiles (PERMANOVA *p* > 0.05; Figure 1B and Table S1), suggesting those group mice showed similar gut microbiota compositions before *Blastocystis* inoculation. After infection for two weeks, all these three groups showed significant differences compared to the baseline (PERMANOVA *p* < 0.05; Figure 1B and Table S1). ST4-colonized mice showed increase in the abundance of *Clostridia* and decrease in the proportion of *Bacteroidia* (Figure 1C and Figure S1E). At the genus level, the *Lachnospiraceae* NK4A136 group, and* Clostridia vadinBB60* group were enriched in ST4-clonized mice (Figure 1D and Figure S1F). Bacteria from these genera are known to be beneficial bacteria, which can produce SCFAs that are a carbon source for intestinal epithelial cells and induction of Treg cells 33, 34. In contrast, ST7 infection caused the reduction in beneficial bacteria *Lachnospiraceae* UCG-001, and* Lactobacillus*, and increased the sulfate-producing bacteria *Desulfovibrio* (Figure 1D and Figure S1F). The enrichment of *Desulfovibrio* was observed in UC patients, suggesting the species of* Desulfovibrio* genus can be involved in colitis development 35, 36. Similarly, the proportion of *Lachnospiraceae* UCG-001, and* Lactobacillus* declined, and *Desulfovibrio* was enriched in control mice (Figure S1F).

Linear discriminant analysis effect size (LEfSe) analysis revealed that the *Deferribacterales*, *Deferribacteraceae*, *Roseburia*, and *Peptococcus* were significantly more abundant in ST4-colonized group compared to the baseline. In contrast, significant increases of *Desulfovibrionales*, *Desulfovibrionaceae*, *Muribaculaceae*, and *Desulfovibrio* were observed in ST7-infected groups (Figure 1E). In general, ST4 colonization was associated with higher proportion of beneficial bacteria, while ST7 infection decreased the abundance of beneficial bacteria and increased sulfate-producing bacteria, which may predispose the host to colitis.

### Effects of *Blastocystis* ST4 and ST7 infection on SCFA production and colonic T helper cells

**Figure 2:**
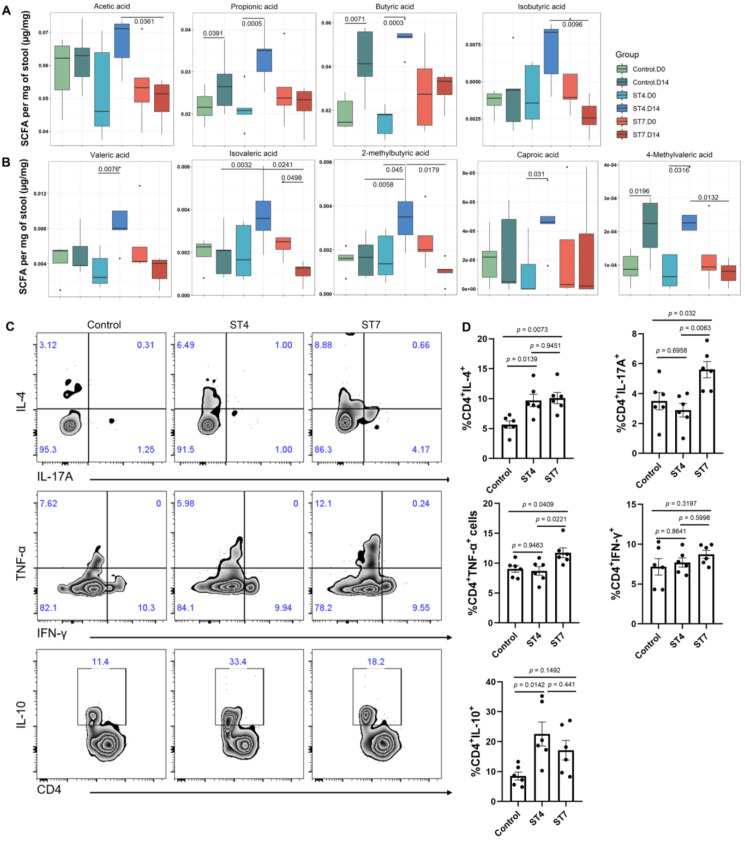
***Blastocystis* ST4 and ST7 infection influence SCFAs production and colonic T helper cells**. (**A**) The concentration of acetic, propionic, butyric, and isobutyric in control, ST4, and ST7 infected mice. (**B**) The concentration of valeric acid, isovaleric, 2-methylbutyric, and caproic acid, and 4-methylvaleric acid. (**C**) Zebra plots show staining for IL-4, IL-17A, TNF-α, IFN-γ and IL-10 within CD4^+^ T cells. (**D**) Bar charts show the percentage of IL-4, IL-17A, TNF-α, IFN-γ and IL-10 expressing CD4^+^ T cells. One-way ANOVA *p*-values adjusted for Tukey's multiple comparisons are shown. Data are representative of two independent experiments and shown as the mean ± SEM.

We next investigated whether *Blastocystis* infection affected the production of SCFAs, which constitute the primary energy sources for colon epithelium and play a role in maintaining intestinal homeostasis 37. Interestingly, ST4-colonized mice showed a significant increase in the majority of SCFAs (Figure 2A-B). However, ST7 did not and instead was associated with reduced isovaleric acid. (Figure 2A-B). As microbiota-derived SCFAs can regulate the adaptive immune response 38, we next investigated whether ST4 and ST7 infection could also influence host immunity (Figure S2A). ST4-colonized mice showed a substantial increase in IL-4 and IL-10-producing CD4^+^ T cells (Figure 2C-D), which is consistent with our previous data 15. Interestingly, the Th1 compartment (expressing TNF-α) and the Th17 subset (expressing IL-17A) were mildly but significantly increased in ST7-infected mice (Figure 2C-D). The effects of ST4 and ST7 infection on host overall health were also investigated. We observed that both ST4 and ST7-infected mice showed comparable effects on body weight change, DAI, colon length, and histopathology scores when compared to control mice (Figure S2B-C). These data suggest that ST4 and ST7 infection did not cause obvious pathological changes, but elicited different immune responses, which may affect the outcome of the host's response to other stimuli.

### Effects of *Blastocystis* ST4 and ST7 infection on the severity of experimental colitis

**Figure 3:**
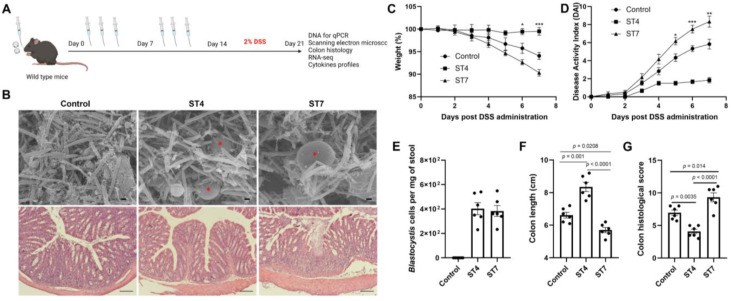
**The effects of *Blastocystis* ST4 and ST7 colonization on subsequent DSS challenge**. (**A**) Study design. (**B**) SEM of colonic and cecum tissues from control, ST4, and ST7 infected mice (upper panel), and *Blastocystis* are indicated with red asterisk (Scale bar = 1 μm). H&E-stained colon sections (lower panel) from the three groups (Scale bar = 100 μm). Weight changes (**C**), and DAI (**D**) in control, ST4, and ST7 infected mice. (**E**) The number of *Blastocystis* cells in ST4 and ST7 infected mice. Colon length (**F**) and histological scores (**G**) in the three groups. Data are representative of two independent experiments and shown as the mean ± SEM. Two-way ANOVA with Dunnett's multiple comparison testing were used for multiple comparisons. *p < 0.05; **p < 0.01; ***p < 0.001.

The gut microbiota is implicated in pathogenesis of IBD 39. Next, we explored the effects of altered gut microbiota caused by *Blastocystis* in the development of experimentally induced colitis. Mice were infected with *Blastocystis* and then subjected to induced colitis through oral DSS administration for 7 days (Figure 3A). The *Blastocystis* colonization status and the number of* Blastocystis* cells was examined (Figure 3B and 3E). We observed that ST7-infected mice treated with 2% DSS showed progressive weight loss and DAI from day 5 to day 7 (Figure 3C-D). In contrast, mice colonized with ST4 did not develop these symptoms (Figure 3C-D). Additionally, ST7 infected mice exhibited shorter colon lengths when compared to non-infected and ST4 colonized mice (Figure 3F). Histological analysis of colon tissues showed severe inflammation with loss of crypts and infiltration of inflammatory cells in ST7 infected mice, whereas ST4 colonized mice were protected from DSS-induced damage to the colon (Figure 3B and 3G). Altogether, priming mice with ST4 and ST7 showed distinct effects on the severity of DSS-induced colitis.

### Effects of *Blastocystis* ST4 and ST7 infection on mucosal gene expression in DSS-induced colitis mice

**Figure 4:**
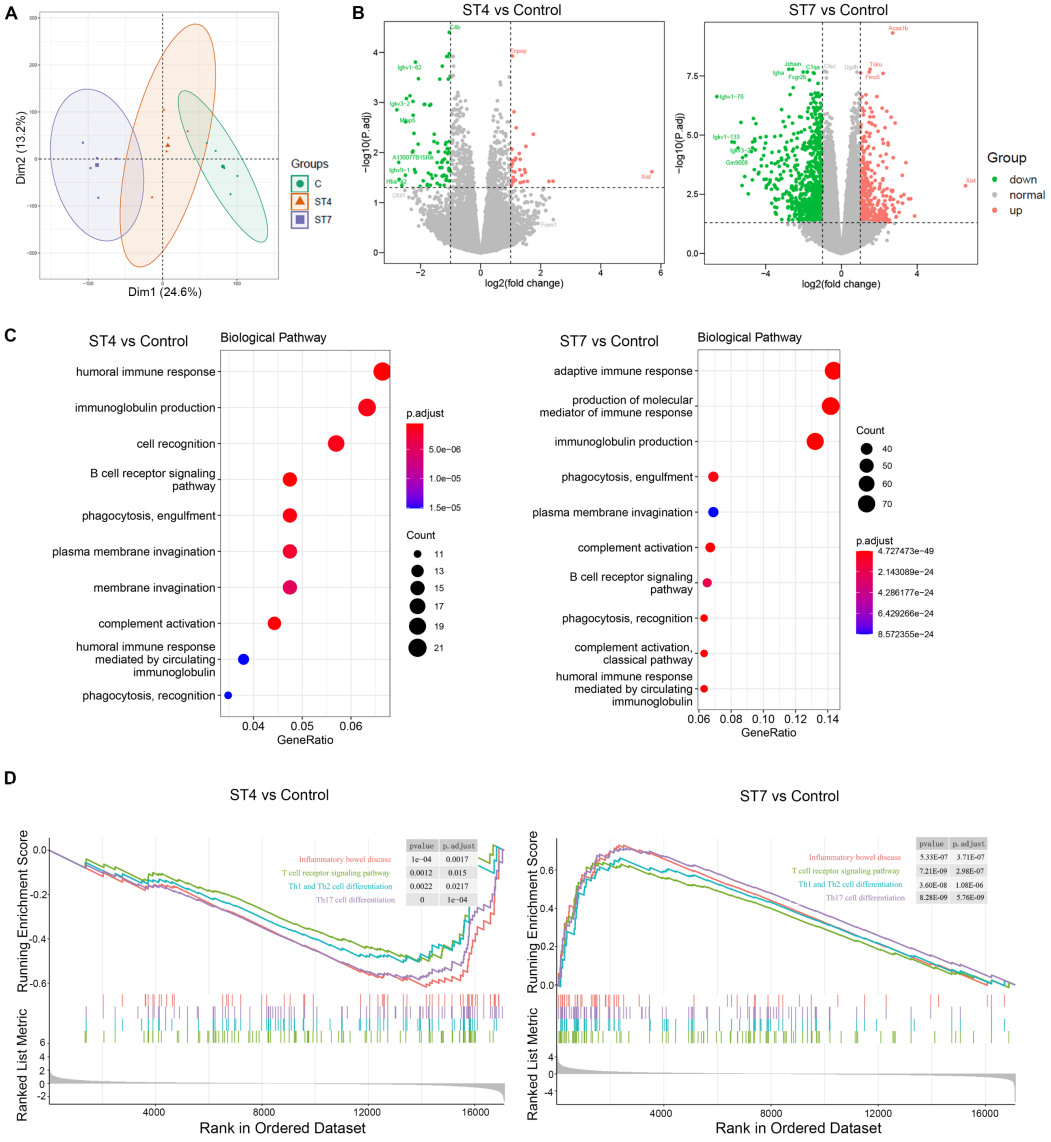
**Gene expression in ST4 and ST7-infected colon tissues after DSS treatment**. (**A**) Principal component analysis (PCA) plot comparing mouse transcriptomes among control, ST4, and ST7 infected mice. (**B**) Volcano plots showing log2 fold change plotted against log of mean normalized expression counts. (**C**) The top 10 most enriched GO terms found in the analysis of DEGs in ST4 vs. Control group, and ST7 vs. Control group. GO terms were ranked by their significance. (**D**) GSEA analysis showed the gene sets of inflammatory bowel disease, T cell receptor signaling pathway, Th1 and Th2 cell differentiation, and Th17 cell differentiation. Graphs depict the enrichment score (y axis) with negative values where gene sets are inhibited, and positive values where they are induced. Each vertical bar on the x axis represents an individual gene within the gene set, and its relative ranking against all genes analyzed. *P* and Padj value is indicated on the graph.

To assess the impact of *Blastocystis* infection on the intestinal epithelium, we analyzed the transcriptional profile of colon tissues from DSS-induced colitis mice. PCA plot showed samples from the same group clustered together (Figure 4A). Pairwise comparisons between the different groups revealed that ST4 colonized mice had 109 differential expressed genes (DEGs) (33 up-regulated and 76 down-regulated) when compared to control mice (Figure 4B). ST7 colonized mice have a far greater number of DEGs with control group than ST4, with 1,142 (391 up-regulated and 751 down-regulated) (Figure 4B). We next analyzed Gene Ontology (GO) terms, which provides a framework and set of concepts for describing the functions of gene products. Interestingly, colonization with ST4 mostly activated humoral immune response and immunoglobulin production pathways (Figure 4C). We observed that ST7 infection was related to the adaptive immune response (Figure 4C). KEGG pathway analysis showed both ST4 and ST7 infection were related to cytokine-cytokine receptor interactions (Figure S3). In addition, other categories of immune-related pathways, such as Th17 cell differentiation and inflammatory bowel diseases, were associated with ST7 infection (Figure S3). To better understand the molecular mechanisms of complex biochemical reactions, we then applied GSEA on the DEGs. We found that ST4 colonization caused a marked reduction in normalized enrichment scores (NESs) for inflammatory bowel disease, T cell receptor signaling pathway, Th1 and Th2 cell differentiation, and Th17 cell differentiation (Figure 4D). In contrast, increased scores for each of these gene sets were found in ST7 infected groups (Figure 4D). In general, the induction seems to be higher for ST7 from the gene ratio which induced more gene profile responses. Mice with prior ST4 colonization are refractory to colitis, associated with protective immune responses, while ST7 infection caused worsening of colitis, related to pro-inflammatory immune responses.

### Effects of *Blastocystis* ST4 and ST7 infection on colonic T helper cells in DSS-induced colitis mice

**Figure 5:**
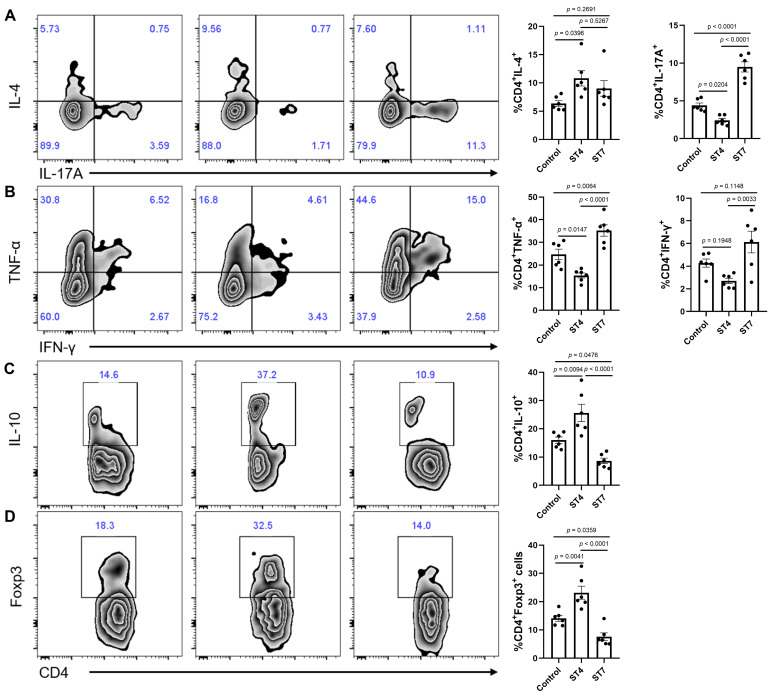
**Colonic immune profiles in control, ST4, and ST7 infected mice after treatment with DSS**. (**A**) Zebra plots and bar charts show staining and the percentage of IL-4, and IL-17A within CD4^+^ T cells. (**B**) Zebra plots and bar charts show staining and the percentage of TNF-α, and IFN-γ within CD4^+^ T cells. Zebra plots and bar charts show staining and the percentage of IL-10 (**C**) and Foxp3 (**D**) within CD4^+^ T cells. Data are representative of two independent experiments and shown as the mean ± SEM. One-way ANOVA *p*-values adjusted for Tukey's multiple comparisons are shown.

T helper cells are thought to play a crucial role in the development of IBD 40. To further investigate whether ST4 and ST7 infection caused changes related to T helper cells, we measured the distribution of Th1, Th2, Th17, and Treg cells within T cells isolated from colonic mucosa. In contrast to ST4 colonized mice, infection with ST7 markedly increased the percentage of pro-inflammatory cytokines IL-17A and TNF-α-producing T cells in the colonic lamina propria (LP) (Figure 5A-B). Concomitantly, the percentage of T cells expressing anti-inflammatory cytokine IL-10 was significantly reduced (Figure 5C). Forkhead box protein 3 (Foxp3) is essential in the development, maintenance, and function of Treg cells, which play a critical role in the maintenance of intestinal homeostasis 41. ST4 colonized mice showed a substantial accumulation of Foxp3^+^ Tregs (Figure 5D). Together, these results suggest that increased susceptibility to colitis after ST7 infection is associated with the induction of pro-inflammatory Th1 and Th17 responses, while ST4 colonization confers resistance to colitis related to protective immune responses.

### Effects of *Blastocystis*-altered gut microbiota on the severity of experimental colitis

**Figure 6:**
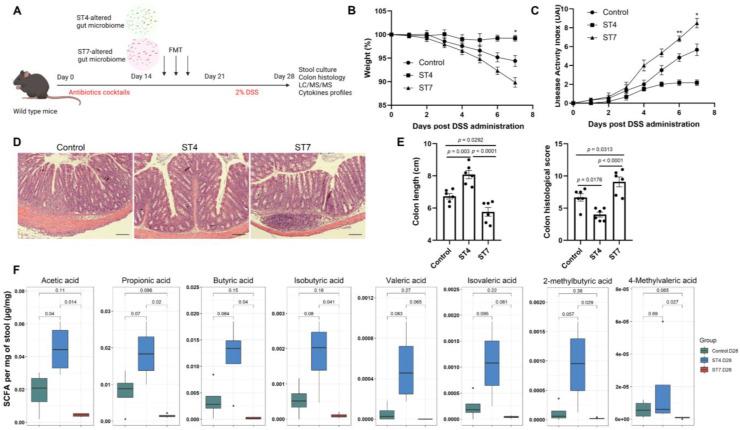
**Effects of *Blastocystis*-altered gut microbiota on the severity of experimental colitis**. (**A**) Study design. Weight changes (**B**) and DAI (**C**) in control, ST4, and ST7 altered microbiota recipient mice. (**D**) H&E-stained colon sections from the three groups (Scale bar = 100 μm). (**E**) colon length and histological scores in the three groups. (**F**) The concentration of acetic, propionic, butyric, and isobutyric, valeric acid, isovaleric, 2-methylbutyric, and caproic acid, and 4-methylvaleric acid in the recipient mice. Data are representative of two independent experiments and shown as the mean ± SEM. One-way ANOVA *p*-values adjusted for Tukey's multiple comparisons are shown (**E**, **F**). Two-way ANOVA with Dunnett's multiple comparison testing were used for multiple comparisons (**B**, **C**). *p < 0.05; **p < 0.01.

We next sought to determine whether the *Blastocystis*-altered microbiota played a direct role in the worsening of, or protection against, DSS colitis. To exclude the effects of *Blastocystis* on the severity of colitis, feces from *Blastocystis* infected mice were frozen at -80°C at least one week before starting the FMT. This treatment ruptures and destroys *Blastocystis* organisms (Figure S4). Recipient mice were treated with antibiotic cocktails to deplete the gut microbiota and then gavaged with ST4 and ST7-altered gut microbiota (Figure 6A). The recipient mice that received FMT from ST4 donor mice had less severe colitis when challenged with DSS, reflected by weight changes and DAI (Figure 6B-C). In contrast, body weight decreased significantly, and DAI tended to be higher in recipients of the ST7-altered microbiota (Figure 6B-C). Additionally, recipients of the ST7-altered microbiota had significantly shorter colon lengths than control recipients, whereas recipients of the ST4-altered microbiota had significantly longer colon lengths (Figure 6E). Histology analysis showed that recipients of the ST4-altered microbiota had significantly lower histological scores than control and ST7-altered microbiota recipients (Figure 6D-E). These findings provide evidence that the *Blastocystis*-altered microbiota, even without *Blastocystis* present, was sufficient to worsen or protect the mice from the DSS-induced colitis.

### Effects of *Blastocystis*-altered gut microbiota on SCFA production and colonic T helper cells

**Figure 7:**
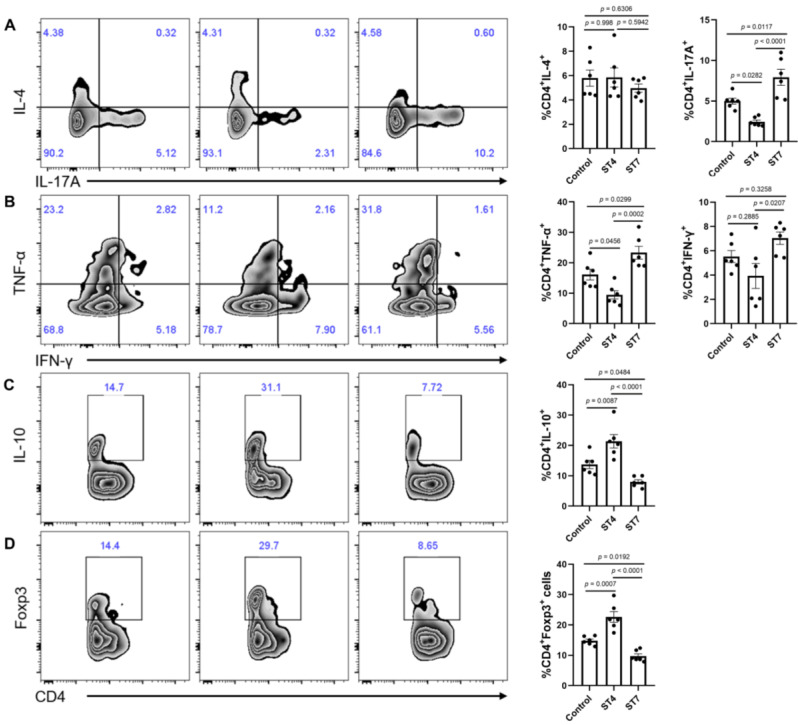
**Colonic immune profiles from recipient mice**. (**A**) Zebra plots and bar charts show staining and the percentage of IL-4, and IL-17A within CD4^+^ T cells. (**B**) Zebra plots and bar charts show staining and the percentage of TNF-α, and IFN-γ within CD4^+^ T cells. Zebra plots and bar charts show staining and the percentage of IL-10 (**C**) and Foxp3 (**D**) within CD4^+^ T cells. Data are representative of two independent experiments and shown as the mean ± SEM. One-way ANOVA *p*-values adjusted for Tukey's multiple comparisons are shown.

To understand mechanistically how ST4 and ST7-altered gut microbiota differentially impact DSS-induced colitis, we measured the concentration of SCFAs among the three groups to determine whether these were associated with the development of colitis. The concentrations of acetic acid, propionic acid, butyric acid, isobutyric acid, 2-methylbutyric acid, and 4-methylvaleric acid in fecal samples of ST4-colonized mice were significantly higher than in ST7-infected mice (Figure 6F). We next sought to investigate whether the *Blastocystis* altered microbiota can modulate the colonic adaptive immune responses. Although there was no significant difference in the percentage of IL-4-producing Th2 cells among the three groups, we observed a substantial increase in IL-10^+^ and Foxp3^+^ Treg cells in the colonic mucosa of ST4-altered microbiota recipients (Figure 7A, C-D). Similarly, we also detected a significant expansion of TNF-α-producing CD4^+^ Th1 cells and IL-17A-producing CD4^+^ Th17 cells after receipt of the ST7-altered microbiota (Figure 7A-B). These data showed that *Blastocystis*-altered gut microbiota play a significant role in the regulation of the pathogenesis of DSS-induced colitis by affecting adaptive immune responses.

## Discussion

Growing evidence suggests that parasitic infections can influence the severity of colitis in a mouse model of IBD 42, 43. Infection with the tapeworm *Hymenolepis diminuta* suppressed experimentally-induced colitis in mice dependent on gut microbiota, suggesting the gut microbiota may influence the outcome of infection and disease 44. In this study, two subtypes were used to investigate the effects of *Blastocystis* on host health. ST4 is more often found in rodents than ST7 which is commonly found in birds 45, suggesting that subtype specificity may play a role in the clinical outcome of *Blastocystis* infection. We demonstrated that mice colonized with *Blastocystis* ST4 showed an increase in the beneficial bacteria *Lachnospiraceae* and *Clostridia vadinBB60* group, which protected the mice when challenged with DSS-induced colitis. However, *Blastocystis* ST7 infection decreased the abundance of beneficial bacteria *Lachnospiraceae* UCG-001, *Lactobacillus*, and *Roseburia*, and increased the level of sulfate-producing bacteria *Desulfovibrio*, which may have exacerbated the severity of the experimental colitis. These findings suggested that *Blastocystis* may serve as a critical component of the anti-colitic or pro-colitic effects in an animal model of colitis through regulating the gut microbiota.

The associations between *Blastocystis* and gut microbiota have been explored in several studies in recent years 46, 47. Although most studies showed *Blastocystis*-carriers are asymptomatic and have healthy gut microbiota, other studies revealed that *Blastocystis* infection was associated with decreased executive functions in humans 48, and fecal dysbiosis in a mouse model 49. It has been suggested that different subtypes exhibited differential effects on the development of the experimental colitis 14, 15, 50. For example, long-term *Blastocystis* ST3 colonization promotes a faster recovery from dinitrobenzene sulfonic acid (DNBS) induced colitis through stabilization of the gut microbiota 50. In contrast, severe colonic damage and ulceration were observed in *Blastocystis* ST7 infected mice when exposed to DSS accompanied by disrupted gut microbiota 14.

In this study, we observed that *Blastocystis* ST4 not only increased the well-known SCFA-producing bacteria but increased the concentration of SCFAs, while *Blastocystis* ST7 did not show such effects, and, to some extent, decreased the level of some SCFAs. SCFAs are the main metabolites produced by the gut microbiota through the anaerobic fermentation of substrates, such as fibers and resistant starch, which play a crucial role in maintaining intestinal homoeostasis 51. Decreased numbers of SCFA-producing bacteria and lower concentrations of fecal SCFAs were also observed in IBD patients 52, 53. SCFAs also regulate immune cells, especially Tregs, by inhibiting histone deacetylase (HDAC) activity or through GPRC receptors, exerting anti-inflammatory effects 54.

High Th1 and Th17 cytokine production in colonic mucosa was related to clinical manifestation of colitis 55. Th2 immune responses can antagonize the production of Th1 cytokines to alleviate colitis 56. Besides, IL-10-producing Treg cells also can suppress pathogenic Th17 cell responses 57. Our data support a mechanism whereby colonization with* Blastocystis* ST4 causes an increased abundance of SCFA-producing bacteria and concentration of SCFAs, which mediates the suppression of colitis by the expansion of Tregs in the colonic mucosa. In contrast, ST7 infection decreased the proportion of beneficial bacteria and SCFAs and increased the sulfate-reducing bacteria, which worsened the severity of colitis through the induction of pro-inflammatory Th1 and Th17 cytokines.

It has been demonstrated that the excretory or secretory products from helminths can inhibit tuft and goblet cells to block the effects of IL-4 and IL-13 in mice 58. On the other hand, secreted products can induce Foxp3^+^ Tregs by activating TGF-β signaling 59. Although live *Blastocystis* cells were excluded from the *Blastocystis-*altered microbiota FMT experiments, we could not exclude the influence of excretory/secretory products on the severity of colitis through FMT. Future directions will explore whether *Blastocystis* can affect gut microbiota composition through secreted products or extracellular vesicles, and whether *Blastocystis* can also modulate the development and progression of inflammatory diseases through excretory/secretory products and other small molecules, such as bile acids and tryptophan metabolites.

FMT is a common therapeutic strategy for restoring disrupted microbiota 60. It has been used as an ordinary standard therapy for recurrent* C. difficile* infection (rCDI) 61, and some trials have reported its potential to alleviate or treat IBD 62. The present consensus for FMT is to exclude *Blastocystis*-positive donor samples 61, 63, though the pathogenicity of *Blastocystis* remains controversial 64. The presence of *Blastocystis*-positive (ST1 and ST3) donor samples for FMT in treatment rCDI did not cause any adverse gastrointestinal symptoms 65, suggesting that certain subtypes can be used for FMT. Since ST1, ST2, and ST3 are very common in humans, donors colonized by one of these 3 subtypes are probably acceptable. Our data show that transplantation of ST4-altered gut microbiota protected mice from later DSS-induced colitis, while ST7 caused converse effects, suggesting the necessity for differentiation of *Blastocystis*-positive donors at the subtype level. Besides, it should be noted that the effects of different strains from the same subtype may also impact intestinal health. For example, the extensive intra-ST7 variability in inducing intestinal barrier dysfunction was observed in our previous study 16, suggesting intra-subtype variability may explain the discrepancy of pathogenicity in *Blastocystis*.

## Conclusions

We report, for the first time, that colonization with *Blastocystis* ST4 exerted a protective effect against the onset of intestinal inflammation by increasing the proportion of beneficial bacteria and SCFAs, and the regulation of colonic CD4 T cells. ST7 infection caused opposite effects on DSS-induced acute colitis. Moreover, transplantation of ST4 and ST7-altered gut microbiota was sufficient to transfer the protective and inflammatory phenotypes respectively. As the murine model is not exactly reflective of the human gut in the case of *Blastocystis* infection, it is necessary to perform, in future studies, human gut microbiome studies to reveal the precise roles of ST4 and ST7 on intestinal inflammation.

## Supplementary Material

Supplementary figures and table.

## Abbreviations

IBD: Inflammatory bowel disease
CD: Crohn's disease
UC: ulcerative colitis
Th: T helper
Treg: T regulatory
DSS: Dextran sulfate sodium
IMDM: Iscove's modified Dulbecco's medium
PBS: Phosphate-buffered saline
DAI: Disease activity index
H&E: Hematoxylin and eosin
SEM: Scanning electron microscopy
QIIME: Quantitative Insights into Microbial Ecology
PERMANOVA: Permutational multivariate analysis of variance
PCoA: Principal co-ordinates analysis
PCA: Principal component analysis
GO: Gene Ontology
KEGG: Kyoto Encyclopedia of Genes and Genomes
GSEA: Gene Set Enrichment Analysis
RPMI: Roswell Park Memorial Institute
FCS: Fetal calf serum
FBS: Fetal Bovine Serum
IL: Interleukin
IFN-γ: Interferon gamma
TNF-α: Tumor necrosis factor alpha
LP: Lamina propria
FMT: Fecal microbiota transplantation
SCFAs: Short-chain fatty acids
HDAC: Histone deacetylase
