# Supplementary material for: Colonization with two different Blastocystis subtypes in DSS-induced colitis mice is associated with strikingly different microbiome and pathological features
Source: Theranostics. 2023 Feb 5;13(3):1165–79. doi: 10.7150/thno.81583 (PMC9925320; doi:10.7150/thno.81583)
Supplement: Supplementary file 1 — Supplementary figures and table. [file thnov13p1165s1.pdf]

Colonization with two different *Blastocystis* subtypes in DSS-induced colitis mice is associated with strikingly different microbiome and pathological features

Lei Deng[1], Lukasz Wojciech[2], Chin Wen Png[2], Dorinda Yan Qin Kioh[3], Yuxiang Gu[3], Thet Tun Aung[2], Benoit Malleret[2], Eric Chun Yong Chan[3], Guangneng Peng[4], Yongliang Zhang[2], Nicholas Robert John Gascoigne[2], and Kevin Shyong Wei Tan[1,*]

[1]Laboratory of Molecular and Cellular Parasitology, Healthy Longevity Translational Research Programme, Department of Microbiology and Immunology, Yong Loo Lin School of Medicine, National University of Singapore, 5 Science Drive 2, Singapore 117545, Singapore

[2]Department of Microbiology and Immunology, Immunology Translational Research Programme, Yong Loo Lin School of Medicine, National University of Singapore, 5 Science Drive 2, Singapore 117545, Singapore

[3]Department of Pharmacy, Faculty of Science, National University of Singapore, 18 Science Drive 4, Singapore 117559, Singapore

[4]The Key Laboratory of Animal Disease and Human Health of Sichuan Province, College of Veterinary Medicine, Sichuan Agricultural University, Chengdu, Sichuan, 611130, China

* Correspondence: mictank@nus.edu.sg

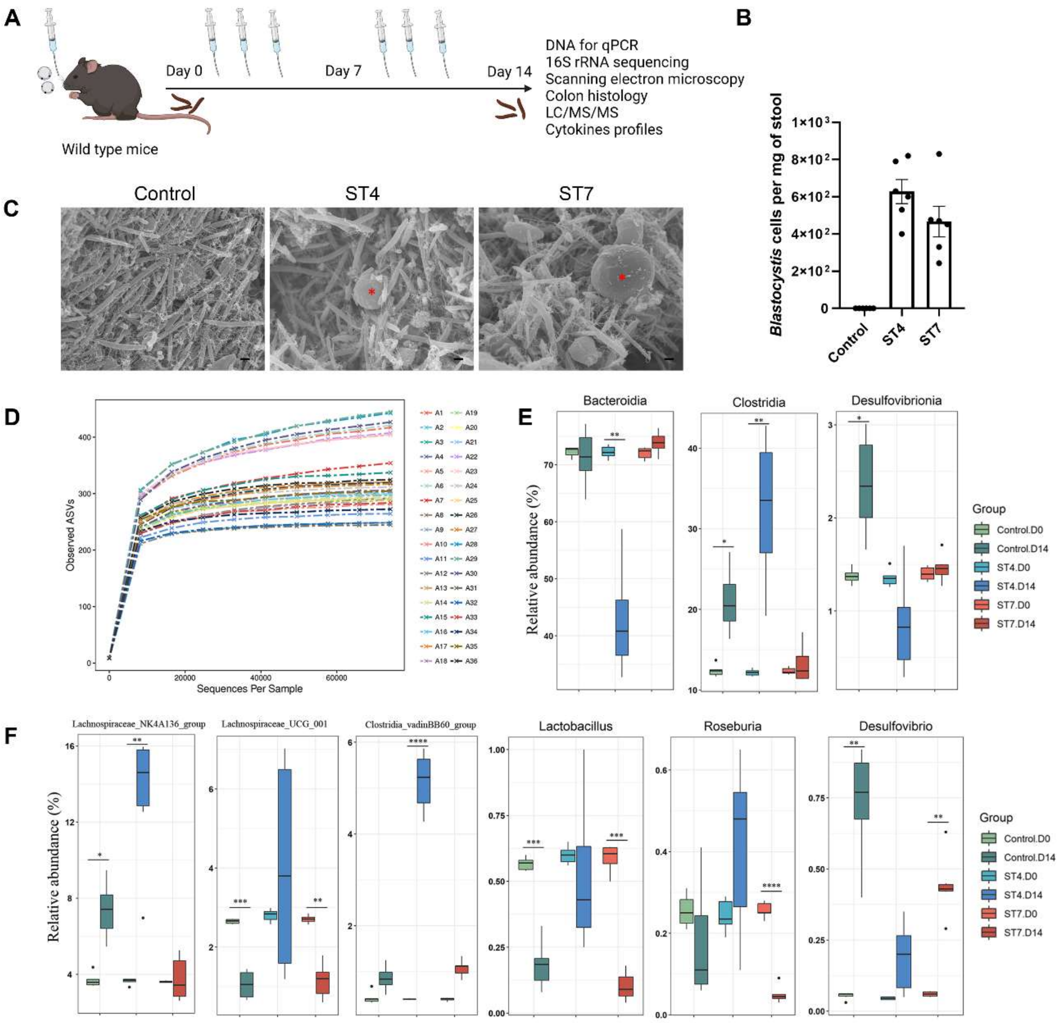

Figure S1. **Effects of *Blastocystis* ST4 and ST7 infection on gut bacterial compositions.** (**A**) Study design. (**B**) The number of *Blastocystis* cells in ST4 and ST7 infected mice. (**C**) SEM of colonic and cecum tissues from control, ST4, and ST7 infected mice, and *Blastocystis* are indicated with a red asterisk (Scale bar = 1 μm). (**D**) Rarefaction curves (threshold is 70,000) showed the depth of sequencing of all the samples was sufficient for microbial analysis. (**E**) Comparison of relative abundances of different taxa at the class level. (**F**) Comparison of relative abundances of different taxa at the genus level. Wilcox rank sum test. *, $P < 0.05$; **, $P < 0.01$; ***, $P < 0.001$; and ****, $P < 0.0001$.

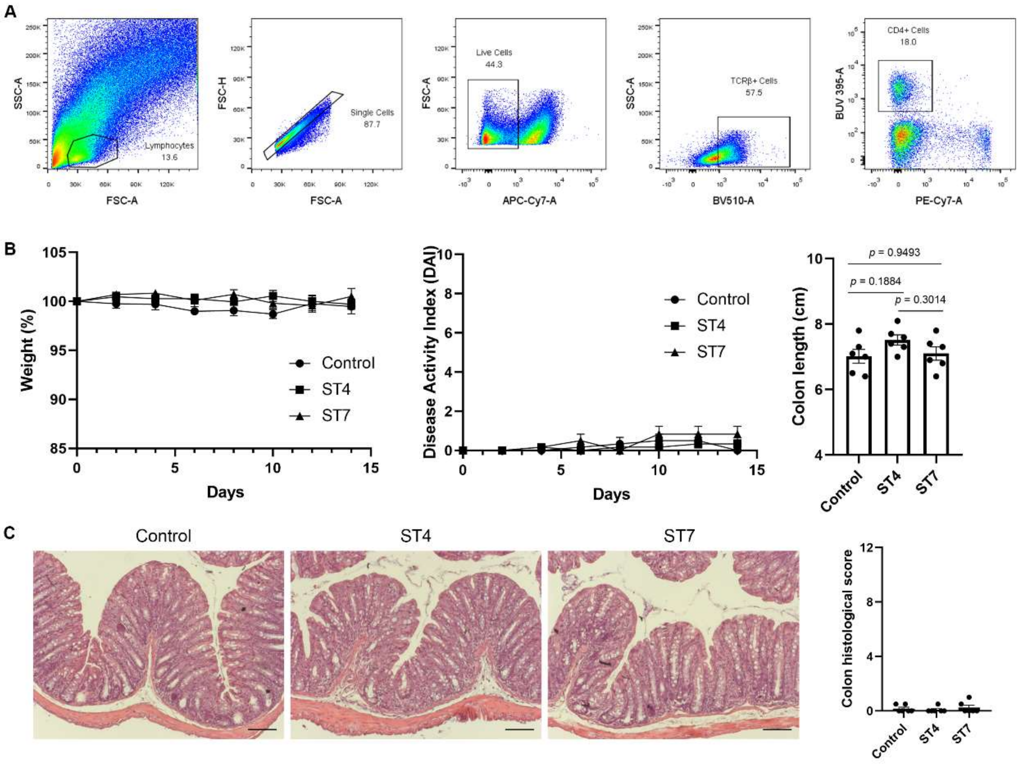

Figure S2. (**A**) Gating strategy of the immune compartments isolated from colonic lamina propria. (**B**) Weight changes, disease activity index (DAI), and colon length from control, ST4, and ST7 infected mice. (**C**) H&E-stained colon sections and histological score from the three groups (Scale bar = 100 µm). One-way ANOVA *p*-values adjusted for Tukey's multiple comparisons are shown.

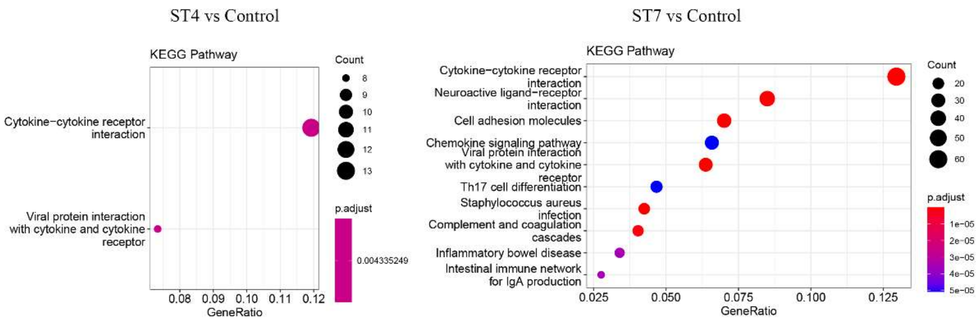

Figure S3. KEGG pathway enrichment analysis of differentially expressed genes (DEGs). The functionally enriched KEGG pathways were found in the analysis of DEGs in ST4 vs. Control group, and ST7 vs. Control group respectively.

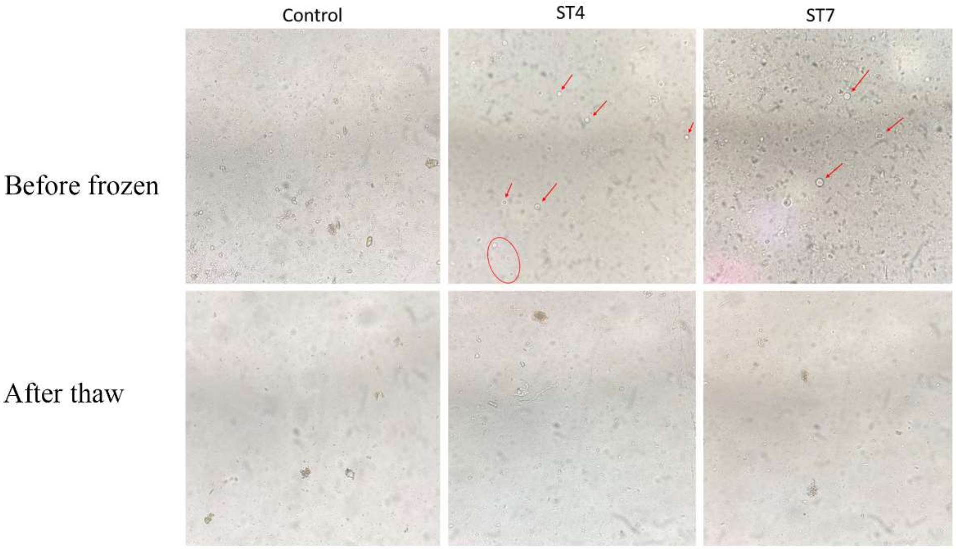

Figure S4. *Blastocystis* in Jones' medium before frozen at – 80 °C and after thaw.

Table S1. PERMANOVA of beta-diversity analysis as measured by Bray-Curtis dissimilarity.

Pairs	Df	SumsOfSqs	F.Model	R2	P.value	P.adjusted
Control_D0 vs ST4_D0	1	0.030743	0.988118	0.089926	0.489	0.489
Control_D0 vs ST7_D0	1	0.018627	1.296218	0.114748	0.082	0.087857
ST4_D0 vs ST7_D0	1	0.030431	1.185969	0.106023	0.063	0.072692
Control_D0 vs Control_D14	1	0.293993	4.655161	0.317647	0.003	0.008333
ST4_D0 vs ST4_D14	1	0.291882	5.778302	0.366218	0.004	0.008333
ST7_D0 vs ST7_D14	1	0.561387	31.34834	0.758152	0.004	0.008333
